# Chronic Liver Disease: A Mimic of Systemic Lupus Erythematosus

**DOI:** 10.7759/cureus.16765

**Published:** 2021-07-30

**Authors:** Gita Verma, Sruthi Kanuru

**Affiliations:** 1 Rheumatology, Carilion Clinic, Roanoke, USA; 2 Rheumatology, Central Arkansas Veterans Healthcare System, Little Rock, USA; 3 Rheumatology, University of Arkansas for Medical Sciences, Little Rock, USA

**Keywords:** lupus, positive ana, liver disease, thrombocytopenia, calciphylaxis, anca

## Abstract

Humoral immune responses can produce autoantibodies against self-cellular proteins and nucleic acids without the presence of autoimmune diseases. Numerous kinds of autoantibodies are detected in liver diseases such as viral hepatitis, alcoholic liver disease (ALD), and nonalcoholic fatty liver disease (NAFLD), where their production could be secondary to hepatocellular inflammation and necrosis. Hence, the presence of an autoantibody does not necessarily indicate the presence of autoimmune disease; nor does it predict its severity and potential response to therapy. In literature, the spectrum and methods of diagnosis of liver disease in lupus are well described. However, chronic liver disease can manifest with signs similar to those in lupus, and it is important to recognize that autoantibodies in patients with chronic liver disease can be seen without the presence of autoimmune rheumatic disease. In this report, we discuss a very interesting case of a middle-aged female with a history of ALD presenting with calciphylaxis, thrombocytopenia, hypocomplementemia, and positive serologies, but without any clinical evidence of autoimmune rheumatic disease.

## Introduction

Though antibodies can be pathognomic in liver conditions like autoimmune hepatitis and primary sclerosing cholangitis, they can also be present in chronic liver conditions like autoimmune hepatitis, viral hepatitis, alcoholic liver disease (ALD), and conditions treated with interferon. There can be multiple reasons for the antigenic stimulation leading to the production of autoantibodies in liver disease: 1. hepatocellular inflammation and necrosis causing antigenic stimulation; 2. treatment with interferon causing oxidative stress [1]. Hence, these antibodies do not help in the diagnosis and predicting the severity of the disease or response to therapy [2].

Chronic liver disease can also have overlapping features of systemic lupus erythematosus (SLE), such as arthralgia, cytopenia, and coagulation abnormalities, when seen with positive serologies and low complements, and can be confused for active SLE. Hypocomplementemia can be seen due to the low production of complement proteins [3].

Systemic lupus erythematosus (SLE) is an autoimmune disorder that can affect multiple organ systems including the kidneys, lungs, joints, bone marrow, and skin. Though certain serologies like antinuclear antibody (ANA), double-stranded DNA (dsDNA), Smith antibody, and low complements seem specific for SLE, they can also be present in the sera of patients with other conditions like viral infections, subacute bacterial endocarditis, malignancy, and chronic liver conditions. Hence, it is important to look for pertinent clinical, laboratory, and immunologic evidence before diagnosing a patient with SLE [4].

## Case presentation

A 68-year-old Caucasian female with a past medical history of pyoderma gangrenosum, glucose-6-phosphate dehydrogenase (G6PD) deficiency, hypothyroidism, anemia, ischemic colitis with gangrenous necrosis, and alcohol dependence was transferred from an outside hospital for evaluation of positive anti-neutrophil cytoplasmic antibody (ANCA), positive ANA, hypocomplementemia, and proteinuria. Two weeks prior to transfer, the patient had been admitted to the outside hospital for sepsis secondary to cellulitis and treated with broad-spectrum antibiotics. The hospital course had been complicated by anemia with a drop in hemoglobin to 5 mg/dL and hypoalbuminemia of 1.1 mg/dL. She had received blood transfusions and albumin infusions. Further laboratory workup had revealed positive p-ANCA and c-ANCA, negative ANA, low complements, and proteinuria. Hence, she was transferred to our institution for further rheumatologic workup and renal biopsy.

Upon presentation, she was afebrile, with a pulse rate of 77, blood pressure of 136/70 mmHg, and respiratory rate of 18/minute. She had ulcerating skin lesions throughout her lower extremity (medial thighs, sacrum, and legs). The complete blood count (CBC) showed WBC of 8.4/cc^3^, hemoglobin of 9.4 mg/dL, hematocrit of 24.2%, and platelet count of 107,000/cc^3^. Our initial differentials were systemic lupus, ANCA vasculitis, and amyloidosis.

However, repeat laboratory workup revealed negative ANCA, ANA of 1:160, mildly positive dsDNA, as well as low C3 and C4. Lactate dehydrogenase (LDH) and haptoglobin were normal. Peripheral smear did not show schistocytes or megakaryocytosis. She had a skin biopsy performed, which showed fat necrosis, focal vascular thrombi, and perivascular calcifications, consistent with calciphylaxis. There was no evidence of interface dermatitis, vasculitis, or amyloidosis. Further testing revealed elevated total bilirubin of 1.8 mg/dl, prothrombin time (PT)/international normalized ratio (INR) of 17.0/1.8, parathyroid hormone (​PTH) 107.1 pg/mL, and 24-hour urine protein of 423 mg.

The patient did not have any clinical evidence of SLE. Serology results (ANA and ANCA) were different from the outside hospital and urine protein was only 423 mg/24 hours, which did not explain the low albumin level of 1.1 mg/dL. Also, other liver function tests were abnormal. Hence, we suspected the patient might have chronic liver disease and ordered further tests, which revealed extremely low CH50 and normal protein C and protein S activity.

**Figure 1:**
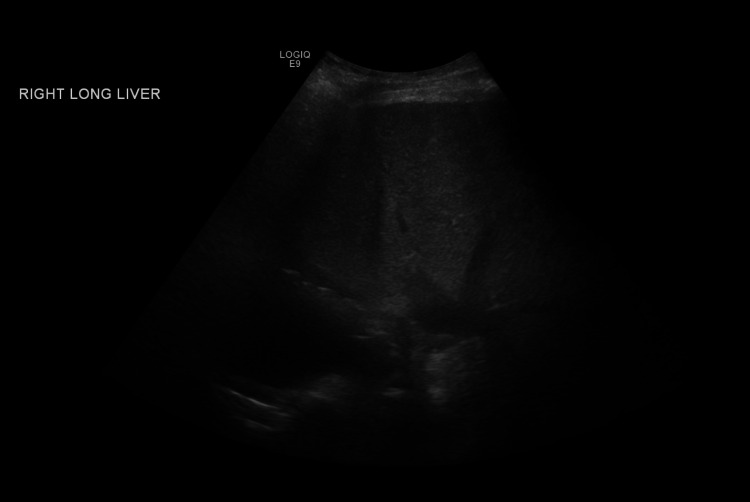
Increase in the parenchymal echogenicity of the liver - view 1

**Figure 2:**
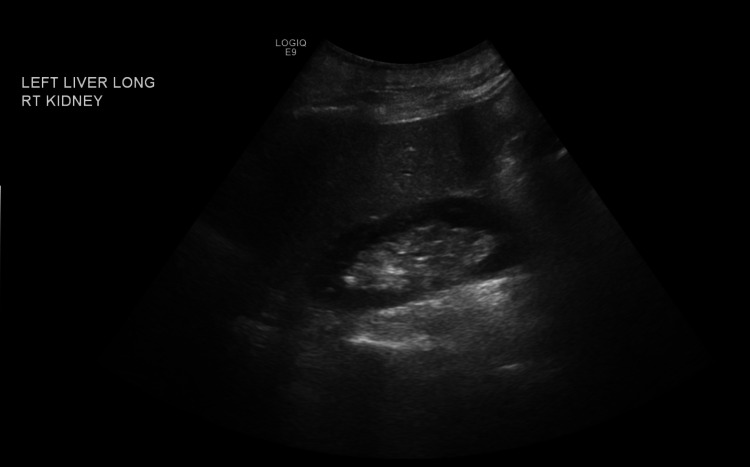
Increase in the parenchymal echogenicity of the liver - view 2

Ultrasound of the liver showed increased parenchymal liver echogenicity, suggestive of parenchymal liver disease with no obstructive/focal lesions (Figures 1, 2). The spleen size was normal.

After consolidating the physical exam findings, laboratory, imaging, and pathology results, we concluded that the patient most likely had chronic liver disease and her hypocomplementemia was due to low production in the liver rather than consumption as seen in active SLE. Her calciphylaxis was attributed to her elevated PTH levels, and she was treated with aggressive wound care and sodium thiosulfate infusions. Her skin lesions and laboratory abnormalities gradually improved without any disease-modifying antirheumatic drugs (DMARDs) or immunosuppressive treatment.

## Discussion

Chronic liver disease can present with nonhepatic systemic manifestations. Patients can have involvement of other organ systems and can have abnormal laboratory findings, which are not limited to liver enzymes and liver function tests. Serological tests show positive antibodies, which are neither specific to the liver and nor help with other diagnoses. Systemic lupus is a multisystemic autoimmune rheumatic disease that can also involve multiple organ systems and positive serologies. On encountering a patient with multiple manifestations, it is important to evaluate the patient carefully before making a diagnosis.

Viral hepatitis, ALD, and nonalcoholic fatty liver disease (NAFLD) are the main etiologies of chronic liver disease. Patients with chronic liver disease can present with various systemic manifestations, such as:

Hematological: anemia, thrombocytopenia [5]

Musculoskeletal: myopathy, arthralgias, and osteopenia [6]

Cutaneous: porphyria cutanea tarda, spider angiomata, telangiectasias, and calciphylaxis [7].

There can be the production of autoantibodies in liver diseases like viral hepatitis, ALD, NAFLD, hepatocellular carcinoma (HCC), and autoimmune liver diseases. ANA, anti-dsDNA, anti-Smith, anti-centromere, and ANCA antibodies, which are seen in rheumatic diseases, can be produced in liver diseases as well. There is antigenic stimulation due to various factors like hepatocellular inflammation, self-cellular proteins, and nucleic acids produced from necrotized tissue [8].

Treatment with interferon also initiates an immune response in patients with chronic Hepatitis C. The antibody response may predict treatment outcomes. It is postulated that oxidative stress also causes antibody production in liver diseases [1]. Albumin infusions can also lead to immune response. The antibodies produced are called naturally occurring antibodies (NOA). They can be either immunoglobulin M (IgM) or IgG isotype. There are two types of NOA. One type of NOA is nonspecific to antigens and has a low affinity. These would help in clearing aged cells. The second type is more specific to albumin with high affinity and would cause immune-related events [9].

In addition to the presence of antibodies, there is an alteration in the complement levels. Based on the etiology of the liver disease and acuity, there can be low, normal, or increased complement levels. In acute or chronic hepatitis, low complement 3 (C3) levels are observed [10]. This could be secondary to decreased production or increased consumption. There is always a correlation between total serum hemolytic complement activity (CH50) and C3. Both levels are either increased or decreased together. However, C4 levels are reduced in all chronic liver disease conditions [11,12].

Though C3 and C4 are the commonly checked complements, other complements that are altered in liver diseases are C2, C5, C6, and C7. C3 and C6 are produced in the liver and also by the cells of the reticuloendothelial system. C2 and C4 are derived from the peritoneum and by mucosal cells in the small intestine. C5 level usually goes along with C3. C7 level is elevated in liver diseases, thereby giving one the impression that it is not produced but catabolized in the liver [11].

## Conclusions

Chronic liver disease can present with systemic manifestations. When combined with antibody testing, it can be misdiagnosed as a rheumatic disease. It is important to recognize that autoantibody testing in patients with liver disease can have limitations and may not contribute to the diagnosis or prognosis of autoimmune rheumatic conditions and should be interpreted carefully, especially because there can be an overlap in the symptoms of autoimmune diseases with those of chronic liver disease. Decreased complement levels are not consistently associated with disease flares and can be due to genetic polymorphism and add little in terms of diagnostic value in patients with chronic liver disease.
